# Antiretroviral Therapy for Prevention of HIV Infection: New Clues From an Animal Model

**DOI:** 10.1371/journal.pmed.0050030

**Published:** 2008-02-05

**Authors:** Myron S Cohen, Angela D. M Kashuba

## Abstract

The authors discuss the implications of a new study in macaques of antiretroviral pre-exposure prophylaxis regimens.

## Background

The introduction of antiretroviral therapy (ART) in the early 1990s profoundly changed the face of HIV infection by improving survival rates [1]. But ART has equal potential for prevention, since it reduces the probability of HIV transmission from an infected person to their sexual partner(s). Although there have been no randomized controlled clinical trials on the subject, antiretroviral drugs are currently used in clinical practice for post-exposure prophylaxis after inadvertent occupational exposure (based on the results of a case control study [2]) or after sexual exposure to the virus [3]. Pre- and post-exposure prophylaxis (PrEP and PEP, respectively) have been used successfully to interrupt transmission of HIV from infected mothers to their babies [4].

Investigators at the United States Centers for Disease Control and Prevention have conducted a series of studies in rhesus macaques to explore antiretroviral prophylaxis. First, they developed a rectal inoculation model using concentrations of simian HIV (SHIV) representative of human exposure [5]. Using this model, the investigators showed that tenofovir disoproxil fumarate (TDF, a nucleotide analogue reverse transcriptase inhibitor) delayed, but did not prevent, acquisition of SHIV in these animals (seven out of eight animals infected over 14 weeks) [6]. A new study in this issue of *PLoS Medicine* by Walid Heneine and colleagues [7] extends earlier observations and will certainly affect the direction of human clinical trials and public health policy.

## The Results

In the new study, macaques were exposed to weekly rectal virus challenges for up to 14 weeks. The authors compared infections observed in 18 untreated macaques to infections in macaques that received a variety of antiretroviral PrEP regimens containing the nucleotide reverse transcriptase inhibitor emtricitabine (FTC) alone or in combination with TDF. With subcutaneous FTC alone (at a human-equivalent dose), four out of six animals became infected. With a combination of oral FTC and TDF at a dose equivalent to Truvada (FTC 200 mg + TDF 300 mg) in humans, two out of six animals became infected. With subcutaneous FTC and a supratherapeutic subcutaneous dose of tenofovir (given either daily or in a two-dose regimen before and after exposure), complete protection from infection was observed (none of the 12 animals became infected).

For animals that became infected during treatment, the investigators noted that infection was delayed, and all animals had blunted acute viremia, suggesting the possibility of reduced immune damage during acute HIV infection [8]. Resistance to FTC was observed in two out of six animals that failed therapy.

This Perspective discusses the following new study published in *PLoS Medicine:*

García-Lerma JG, Otten RA, Qari SH, Jackson E, Cong M, et al. (2008) Prevention of rectal SHIV transmission in macaques by daily or intermittent prophylaxis with emtricitabine and tenofovir. PLoS Med 5(2): e28. doi:10.1371/journal.pmed.0050028

Using a repeat-exposure macaque model, Walid Heneine and colleagues find that pre-exposure prophylaxis with combination antiretroviral drugs provides protection against rectal challenge with a SHIV virus.

## The Implications

These and earlier animal studies have provided the basis for human clinical trials with PrEP. The observation of FTC resistance during therapy emphasizes the risk of PrEP to the individual and the community. PrEP continued in the face of unrecognized infection might be expected to promote replication of a resistant variant, which could be transmitted widely [9]. Tenofovir and FTC resistance are common in populations receiving ART, including in sub-Saharan Africa [10]. In addition, clade C HIV (predominant in sub-Saharan Africa) may be more susceptible to the evolution of a tenofovir resistance mutation (the K65R mutation) [11].

## Strengths and Limitations of the New Study

This new report [7] represents the culmination of a series of recent studies specifically designed to guide and inform human clinical PrEP trials [5,6]. In addition, the new study shows protection from SHIV by intermittent dosing with tenofovir and FTC, a regimen that is closer to true PrEP than continuous daily dosing.

The study had four weaknesses. First, it included only small numbers of animals. Second, nine out of the 18 controls used were historical in nature. Third, FTC and tenofovir doses chosen as human-equivalent were based on first-dose pharmacokinetics in a limited number of animals, and they represent higher drug exposures than seen in humans (FTC and tenofovir areas under the concentration-time curves in macaques were approximately 30% and 40% higher, respectively, than exposures in humans [12]). In addition, intracellular pharmacokinetics of the active agents also differ between macaques and humans [13–15]. Finally, complete protection from HIV acquisition was only observed with a subcutaneous tenofovir dose that provided concentrations greater than can be achieved with oral therapy [16].

## The Future

These results highlight an exciting and potentially important use of ART to prevent sexual transmission of HIV [3], and offer further support for human clinical trials in progress or planned. Optimistic modeling experiments suggest an important role for PrEP in HIV prevention [17]. But the application of PrEP highlights a unique tension between prevention and treatment; widespread usage of ART for prevention in communities where ART for treatment is still being rationed might cause conflict [18]. Also, PrEP has the potential to accelerate transmitted drug resistance [9], thereby limiting the utility of drugs critical to combination ART. Human PrEP trials must address these concerns. One PrEP safety trial has been completed in women at high risk of acquiring HIV in Africa [19]; other current trials designed to measure PrEP safety and efficacy are summarized in Table 1. These PrEP trials will shine a light on the potential of ART for prevention, and help physicians to think more broadly about the public health implications of these life-saving drugs.

**Table 1:**
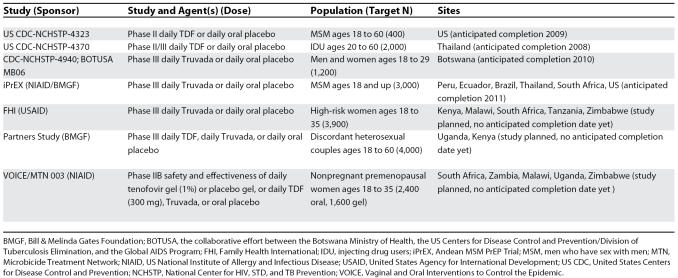
Current and Proposed Pre-Exposure Prophylaxis Trials, October 2007

## Abbreviations

ART: antiretroviral therapy
FTC: emtricitabine
PrEP: pre-exposure prophylaxis
SHIV: simian HIV
TDF: tenofovir disoproxil fumarate
